# West Nile Virus Pilot Screening in Field-Collected *Aedes japonicus* (Theobald, 1901): An Update of Species Distribution in Poland, 2025

**DOI:** 10.3390/v17111515

**Published:** 2025-11-19

**Authors:** Paweł Niemiec, Jowita Samanta Niczyporuk, Wojciech Kozdruń, Agnieszka Stolarek, Łukasz Mielczarek, Kamil Słomczyński, Kacper Barszcz, Paweł Kuziora, Grzegorz Jarosiewicz, Alicja Jarosz, Andrzej Józef Woźnica, Grzegorz Zaleśny, Mariusz Gwardjan, Gabriela Ochała-Gierek, Marcin Gierek

**Affiliations:** 1 Department of Biochemistry and Medical Genetics, School of Health Sciences, Medical University of Silesia in Katowice, 40-752 Katowice, Poland; pniemiec@sum.edu.pl; 2 Department of Virology and Animal Viral Diseases, National Veterinary Research Institute, 24-100 Puławy, Poland; jowita.niczyporuk@piwet.pulawy.pl (J.S.N.); wkozdrun@piwet.pulawy.pl (W.K.); agnieszka.stolarek@piwet.pulawy.pl (A.S.); 3 Krakow Municipal Greenspace Authority, 30-059 Kraków, Poland; lmielczarek@zzm.krakow.pl; 4 Student at Department of Invertebrate Zoology and Hydrobiology, Faculty of Biology and Environmental Protection, University of Lodz, 90-136 Łódź, Poland; kamil.slomczynski@edu.uni.lodz.pl; 5 Independent Researcher, 25-437 Kielce, Poland; kacperigor2137@gmail.com; 6 Independent Researcher, 37-403 Jastkowice, Poland; pawelkuziora1@gmail.com; 7 Independent Researcher, 34-300 Żywiec, Poland; grzesjar@wp.pl; 8 Institute of Biology, Wrocław University of Environmental and Life Sciences, 50-375 Wrocław, Poland; grzegorz.zalesny@upwr.edu.pl; 9 Regional Veterinary Inspectorate in Kielce, 25-116 Kielce, Poland; m.gwardjan@wiw.kielce.pl; 10 Gierek Clinic, 43-450 Ustroń, Poland; g.ochala@wp.pl (G.O.-G.); gierek@wp.pl (M.G.)

**Keywords:** Asian bush mosquito, *Aedes japonicus*, West Nile virus, invasive species, WNV transmission

## Abstract

(1) Background: The Asian bush mosquito *Aedes japonicus* is an invasive species in Europe, including Poland. Given its laboratory-confirmed competence for West Nile virus (WNV) transmission and its detection as a WNV vector in field-collected mosquitoes, this study investigated whether Polish *Aedes japonicus* harbor WNV and aimed to update knowledge on its distribution in Poland. (2) Methods: In September 2024, 137 adult *Aedes japonicus* were collected from three suburban sites in Poland (Kielce, Mikołów, Kraków). Specimens were screened for WNV using RT-PCR and rRT-PCR. Additionally, unpublished records of *Aedes japonicus* were compiled to update the species’ distribution. (3) Results: No WNV genetic material was detected in field-collected mosquitoes in Poland. By 2025, *Aedes japonicus* had been recorded in half of Polish voivodeships, with most observations in Małopolskie, Śląskie, and Łódzkie. The largest adult populations occurred in Kielce. Ecological traits in Poland matched European and US data, including larval development in artificial containers, preference for suburban and forested habitats, and peak adult activity in late summer. (4) Conclusions: Although WNV was not detected, the rapid spread of *Aedes japonicus* in Poland underlines the need for continued monitoring of its distribution, population dynamics, and potential role in WNV transmission.

## 1. Introduction

The Asian bush mosquito *Aedes japonicus* (Theobald, 1901) is a member of the family Culicidae (Meigen, 1818), native to East Asia (Japan, the Korean Peninsula, Taiwan, southern China, and southeastern Siberia) [1]. During the final decade of the 20th century, this species was recorded for the first time outside its native range, in Oceania [2], and a few years later in the United States (US), where it is currently widespread [3]. The first European record dates to the year 2000 in France [4]. Over the past twenty-five years, the species has considerably expanded its range across Europe. Its presence was reported in Belgium in 2002 [5], in Switzerland and Germany in 2008 [6], in Austria and Slovenia in 2011 [7], in Hungary and the Netherlands in 2012 [8,9], in Croatia in 2013 [10], in Italy and the Principality of Liechtenstein in 2015 [8,11], and in Bosnia and Herzegovina in 2017 [12]. Subsequent detections include Spain and Serbia in 2018 [12,13], Slovakia and Romania in 2020 [14,15], and the Czech Republic in 2021 [16]. In recent years, *Aedes japonicus* has also become established in Poland. The first records, originating from Kielce (Świętokrzyskie voivodeship) in 2022, were uploaded to the citizen science platform iNaturalist by one of the authors of the present study [17]. Publications documenting its occurrence in Poland appeared in 2024 [18,19] and demonstrate that its distribution encompasses a substantial part of southern Poland.

The primary driver of the expansion of the Asian bush mosquito is considered to be the international transport of used tires [20,21]. The introduction success of *Ae. japonicus* in Europe and the United States results from several overlapping factors. First, it is a temperate-zone species, highly tolerant of low temperatures, capable of overwintering in temperate regions in the egg stage, or less commonly in the larval stage [22]. Although its larvae prefer clean, stagnant water, compared with native mosquito species *Aedes japonicus* is less sensitive to organic pollution, allowing it to colonize less favorable environments [4,5,22]. Another important factor is the broad range of larval habitats, spanning natural sites such as puddles, tree holes, or water-filled rock crevices, as well as anthropogenic water containers, including microhabitats such as rainwater tanks, ornamental ponds, uncovered tire stockpiles, flowerpots, watering cans, cemetery vases, animal drinking troughs, and other containers that can accumulate even small amounts of water [22]. The exploitation of such artificial microhabitats increases the drought resistance of *Ae. japonicus* and facilitates its establishment in urbanized areas. Equally relevant are its rapid developmental cycle—limited under optimal conditions to approximately two weeks—the production of multiple generations per year, long period of seasonal activity (in European conditions from April to November), and its diurnal activity, which increases the likelihood of contact with humans. The ecological flexibility and broad tolerance of *Ae. japonicus* make it an exceptionally adaptive invasive species in temperate regions, posing a significant threat both to local biodiversity, owing to its potential to displace other species [23], and to public health. The ease with which *Aedes japonicus* colonizes new areas and habitats underscores the necessity for continuous monitoring of its occurrence and for regularly updating distributional records in regions where it has been detected.

*Aedes japonicus* exhibits vector competence for a wide range of arboviruses, as we have described in detail in our previous study [18]. In natural conditions, however, direct evidence of vector involvement in the transmission of a given pathogen is its detection in field-collected mosquitoes. Among the pathogens present in Europe, the West Nile virus (WNV) has been identified in field-collected specimens of *Aedes japonicus*. It should be noted, however, that all documented cases of WNV carriage by *Ae. japonicus* originate from the United States [24,25]. In Europe, local outbreaks of West Nile fever (WNF) overlap with the current distribution range of *Aedes japonicus* [26,27], yet its role as a WNV host has not been documented to date.

West Nile virus has been circulating in Polish ecosystems for years. Circulation occurs between mosquitoes and birds, while humans and horses are regarded as dead-end hosts. The presence of WNV antibodies has been demonstrated in birds, horses, and humans in Poland [28,29,30,31]. Viral penetration among wild birds appears to be high in certain cases, as evidenced by research conducted by members of our team on material collected in Warsaw in autumn 2022 [29]. Of 99 dead birds from 10 species, WNV was detected by reverse transcription polymerase chain reaction (RT-PCR) in 17.17% of brain homogenate samples. All positive cases were restricted to the hooded crow (*Corvus corone cornix*, Linnaeus 1758), in which the infection rate reached 34.69% in the examined sample [29]. In a study involving human material, the presence of anti-WNV IgG antibodies was investigated in forestry workers—a professional group particularly exposed to mosquito bites—from the Świętokrzyskie and Podlaskie Voivodeships [31]. Antibody prevalence was found to be 28.85% and 34.14%, respectively. In another study, among 42 Polish patients with neurological symptoms characteristic of meningitis, lymphocytic meningitis, and tick-borne encephalitis, anti-WNV antibodies were detected in 33.33% of cases using enzyme-linked immunosorbent assay (ELISA) method [28]. Although the first case of West Nile fever in a human in Poland was recorded in 2005, in a febrile woman from the Podlaskie Voivodeship with no history of travel abroad, however the genetic material of WNV has not been isolated [32], the true scale of the problem remains unknown, given that most infections in humans are asymptomatic or present only with mild symptoms. Nevertheless, there is no doubt that outbreaks of West Nile fever occur in Poland on an annual basis. According to data from the European Centre for Disease Prevention and Control (ECDC), between 2 April and 25 November 2024, six outbreaks in equids and two in birds were confirmed in Poland [33]. These figures are certainly incomplete.

In light of the ongoing expansion of *Ae. japonicus* in Poland and the established presence of WNV in Polish ecosystems, the primary objective of the present study was to investigate whether *Aedes japonicus* specimens collected in Poland are hosts of West Nile virus. A secondary objective was to update the distribution of *Ae. japonicus* in Poland, based on our own data as well as verified observations published online on the citizen science platform iNaturalist by volunteer naturalists.

## 2. Materials and Methods

### 2.1. Mosquito Collection and Location Characteristics

Mosquito collections for West Nile virus pilot screening were conducted in September 2024 in the vicinity of Kielce (Świętokrzyskie Voivodeship), Mikołów (Śląskie Voivodeship), and Kraków (Małopolskie Voivodeship), once per week. The collection sites were selected based on locations known to the authors as habitats of the species. Four collectors (P.N., K.B., M.G., and Ł.M.) participated in the field collections, with detailed information provided in Table 1. Specimens were collected using an entomological net or by the hand-picking method. All collections were conducted during daytime. In total, 137 *Ae. japonicus* specimens were obtained for analysis across all sites (Table 1).

In the Kielce area, two sampling sites were established for collection. During the first week of September, mosquitoes were collected in the Bukówka Forest at the foot of Mount Telegraf (DB73). In the second week, specimens were obtained from the Barcza Reserve, a geological nature reserve located in the municipality of Zagnańsk, approximately 15 km northeast of central Kielce (DB84). Both Kielce sites were situated in suburban forested areas, characterized by periodically waterlogged terrain (Kielce) or the presence of water bodies (a flooded quarry within the Barcza Reserve).

The Mikołów site corresponds to the location where the first *Ae. japonicus* specimens in Poland confirmed by sequencing were collected in 2023 (CA56) [18]. For the present study, all specimens from Mikołów were obtained from a rainwater barrel and the walls of a residential building directly adjacent to a suburban forest covering Kamionka Hill. Although this forest lacks aquatic habitats suitable for mosquito development, the surrounding single-family homes and properties provide abundant artificial water-holding containers serving as substitute larval habitats.

In Kraków, mosquitoes were collected from a riparian forest along the Dłubnia River in the Nowa Huta district (DA34). This site directly bordered the Wanda Allotment Garden (Polish: *Rodzinny Ogród Działkowy Wanda*). Allotment gardens are widespread in Poland, consisting of small recreational plots equipped with basic gardening infrastructure, which allow urban residents to spend leisure time near their homes. In the urban context, allotment gardens—alongside cemeteries and waste disposal sites—represent one of the primary sources of artificial water containers (e.g., water barrels, watering cans, rainwater tanks) that may serve as breeding habitats for mosquitoes.

### 2.2. Aedes japonicus Species Identification

**Figure 1:**
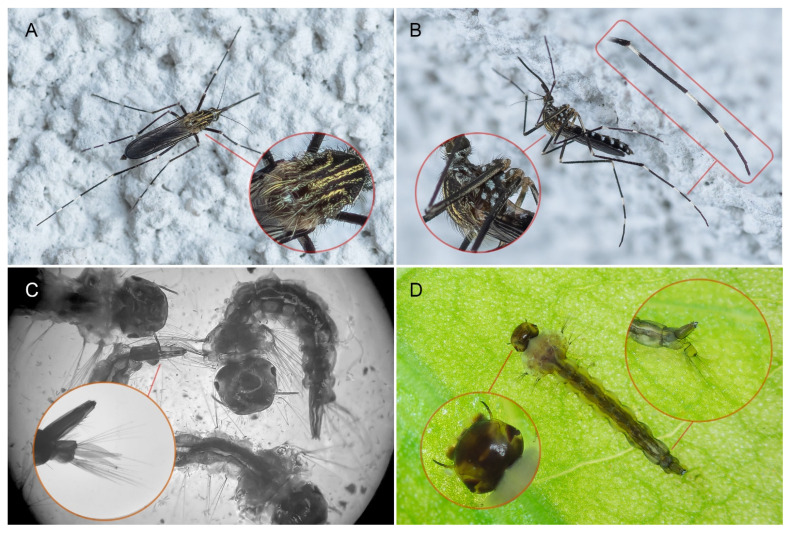
Characteristic features of *Aedes japonicus*. Adult female, top (**A**) and side (**B**) views (P.N.). (**C**) Larvae in microscopic photos (G.J.), and (**D**) from the field (P.K.).

The identification of the collected insects based on the literature [34,35]. The characteristic features of adult *Ae. japonicus* are distinctive patterns of black and white setulae (hairs) on their pleural (sides of the thorax) and mesonotal (dorsum of the thorax) areas, with golden-yellow lyre-shaped stripes on the scutum (the main dorsal part of the thorax). The anterior dorsocentral stripes extend well visibly beyond half of the scutum. The hind tarsomeres (last segments) of the third pair of legs have three whitish cross-bands (Figure 1A,B). Of the 139 mosquitoes specimens collected to screening for the presence of WNV, only two did not belong to the *Ae. japonicus* species and were rejected from the study.

The larval stages (Figure 1C,D) were identified on the morphological features given by Becker et al., 2020 [36]. Larvae of *Ae. japonicus* exhibit uncovered ventral part of anal segment with the saddle seta (1-X) inserted within the saddle and they lack the precratal setae (4-X). The siphon has 14–28 pecten teeth with the distal 1–4 detached. Both the inner (5-C) and median (6-C) frontal setae are multibranched and aligned nearly in a straight line close to the anterior edge of the frontoclypeus [36].

### 2.3. Screening for the Presence of WNV

Insects collected in Kielce were processed immediately after capture. Samples were placed in containers with dry ice and, following transport, transferred to an ultra-low temperature freezer (−80 °C). Subsequent samples from Mikołów and Kraków were deposited directly into the ultra-low temperature freezer. All material was transported on dry ice to the National Veterinary Research Institute in Puławy, Poland, National Reference Laboratory for West Nile virus, where analyses for the presence of West Nile virus were performed.

Mosquito samples collected at each location and date were homogenized separately. A total of 14 samples were prepared, each containing approximately 10 mosquitoes (from 6 to 12 individuals, depending on the number of mosquitoes collected on a given day, Table 1). Viral RNA was extracted from 200 µL of homogenates obtained from mosquitoes using the viral RNA Mini Kit (Qiagen, Hilden, Germany) following the manufacturer’s protocol. In the next step, viral RNA was suspended in a RNase inhibitor buffer (Life Science, St. Petersburg, FL, USA). RNA concentrations were determined using a spectrophotometer (Biorad, Hercules, CA, USA). Viral RNA was kept at −80 °C before use.

The WNV infectious strain (Gene bank accession: OP804520) isolated from the brain of an infected *Corvus corone cornix* was provided by the Reference Laboratory for WNV (National Veterinary Research Institute, Puławy, Poland). Two negative controls were used during RT-PCR and rRT-PCR, and were acquired from a chicken embryo fibroblast CEF, SPF cultures.

Two oligonucleotide primers were set for RT-PCR amplification. Primers for RT-PCR were designed to target the conserved sequence 3′NCR (non-coding region, GeneBank Accession number: DQ211652) and were as follows: WNVF (sense primer): 5′ AAA GCC CAA TGT CAG ACC AC 3′ and antisense primer WNVR: 5′ TAG TCC TTT CGC CCT GGT TA 3′, as indicated by Niczyporuk [37]. The primers and protocol used for the rRT-PCR technique were based on the protocol published by Eiden [38]. The RT-PCR method described by Niczyporuk [37] was used to detect WNV genome. Following the protocol for confirming the molecular detection method of WNV rRT-PCR was conducted [38].

### 2.4. An Update on the Occurrence Range of Aedes japonicus in Poland

The first studies documenting the presence of *Ae. japonicus* in Poland were published in 2024 and included data from 24 localities [18,19]. The present update of the species’ distribution is based on new, previously unpublished observations covering the years 2023–2025. The collected data included the date of observation, geographic coordinates (latitude and longitude), and the number of individuals recorded at various developmental stages. Geographic coordinates were also used to determine the type of land use at the sites where the species was detected. Habitats were classified according to land use categories as follows: forest/schrub (forests, nature reserves, urban parks), rural (agricultural areas and villages), suburban (single-family housing districts, housing estates, allotment gardens, cemeteries, wastelands), and urban (city centers, industrial areas).

The analysis incorporated both the authors’ own records and observations made publicly available on the citizen science platform iNaturalist (as part of the *Mosquito Vectors* project) by volunteer naturalists. Only iNaturalist observations that were unambiguously identified based on photographs of adult mosquitoes exhibiting the key characteristics of *Ae. Japonicus*, and that contained precise geographic coordinates within Poland were included. If an iNaturalist contributor did not provide information on the number of observed mosquitoes, the observation was assumed to represent a single individual.

A total of 37 records were excluded from the analysis, including 32 from iNaturalist. Exclusion criteria for iNaturalist observations were: location outside Poland, insufficient photographic quality preventing reliable identification, lack of precise geographic coordinates, or prior acquisition of the same data directly from the observer. Additionally, five records provided to the authors by non-iNaturalist observers were excluded due to missing geographic coordinates.

Based on geographic coordinates, the colonized grid squares were determined using the Universal Transverse Mercator (UTM) system with a resolution of 10 × 10 km. These data were used to generate an updated distribution map of *Aedes japonicus* in Poland. The map was produced with the software MapaUTM ver. 6 [39].

## 3. Results

### 3.1. Capture of Mosquitoes for WNV Screening

In September 2024, *Aedes japonicus* was the dominant mosquito species at both sites in Kielce (Kielce Bukówka and Barcza Reserve). At the Kielce Bukówka site, hundreds of mosquitoes were observed actively attacking the collectors (P.N., K.B.). Of the 74 mosquitoes captured, 73 were identified as *Ae. japonicus* and one belonged to the *Culex pipiens* complex. At the Barcza Reserve site, *Ae. japonicus* was less abundant; however, the 20 individuals captured represented only a small fraction of the mosquitoes observed. Here as well, *Ae. japonicus* was the dominant species, as no other mosquito species were recorded on the collection day. In Mikołów, *Aedes japonicus* was less numerous compared to the Kielce sites. During each week of observations, the majority (80–100%) of mosquitoes were captured, although a few individuals escaped. The species was dominant at this site, with only a single *Culex pipiens* complex individual recorded in one sample, which was subsequently released. In Kraków, *Aedes japonicus* was also less abundant than at the Kielce sites. A sample of 12 mosquitoes represented approximately 50% of the individuals observed on the collection day. No other mosquito species were recorded at the Kraków site.

### 3.2. West Nile Virus Screening in Field-Collected Aedes japonicus

No genetic material of West Nile virus was detected in homogenates of *Ae. japonicus* collected in locations in Kielce, Mikołów and Kraków.

### 3.3. An Update of Aedes japonicus Distribution in Poland, 2025

In total, data from 67 unpublished observations of *Aedes japonicus* in Poland were collected, including 47 records obtained by the authors and 20 records from the iNaturalist platform (Supplementary Table S1). Most observations concerned adult mosquitoes. Additionally, nine observations of the larval stage were documented, with adult mosquitoes present in four of those cases.

The highest number of new records originated from the Małopolskie Voivodeship (21 observations, 31.34%), followed by Śląskie (n = 17, 25.37%) and Łódzkie (n = 14, 20.90%). The species was also recorded in Podkarpackie (7 observations, 10.45%), Świętokrzyskie (6 observations, 9.00%), Dolnośląskie (1 observation, 1.49%), and Wielkopolskie (1 observation, 1.49%), the latter representing the first record of *Ae. japonicus* in this region (Table 2).

With respect to land use types where adult mosquitoes were recorded, the majority of observations originated from suburban areas (n = 28, 45.16%), followed by forest/shrub habitats (n = 15, 24.19%), urban areas (n = 10, 16.13%), and rural areas (n = 9, 14.52%). It should be noted, however, that habitats classified as suburban in this study often bordered green areas (forests, nature reserves, ecological sites) or were situated within large metropolitan agglomerations/highly urbanized city zones, similar to some of the forest/shrub habitats (e.g., urban parks, urban forests, nature reserves). The sites where mosquitoes were observed frequently exhibited mixed land use characteristics and were subject to dynamic changes and ecological succession over time.

**Figure 2:**
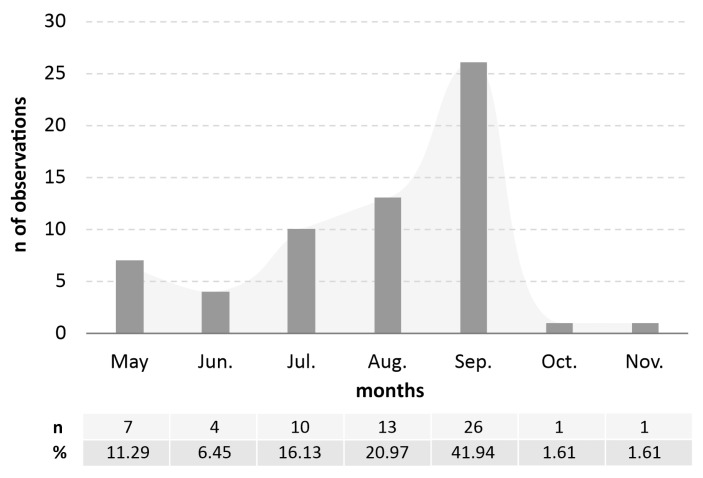
Seasonal activity of adult *Aedes japonicus* in Poland (unpublished observations of adults from 2023–2025).

The number of adult individuals recorded per single observation ranged from one (36 observations, 58.06%), to a few (n = 13, 20.97%), a dozen or so (n = 5, 8.06%), or several dozen individuals (n = 7, 11.29%). Only in one case, at a site in the Świętokrzyskie Voivodeship (Kielce, Bukówka district, 1 September 2024), did the abundance of adult *Ae. japonicus* exceed several hundred (1.51%) (Supplementary Table S1). Over the years of observation (2023–2025), adults were recorded between May and November. The largest number of observations occurred in September (n = 26, 41.94%). The frequency of adult observations by month is presented in Figure 2.

Larvae were observed in suburban, rural, and forested areas (Table 2). The larval habitats consisted predominantly of human-made (artificial) containers, such as plastic buckets or rainwater barrels (n = 8) and a swimming pool (n = 1). Only at one site were larvae also detected outside an anthropogenic container, in water accumulated within a nearby tree cavity. The number of *Ae. japonicus* larvae recorded per observation ranged from several to several hundred (Supplementary Table S1). Larval presence was documented between April and September, with the highest number of records in August (n = 3, 33.33%) and September (n = 3, 33.33%).

**Figure 3:**
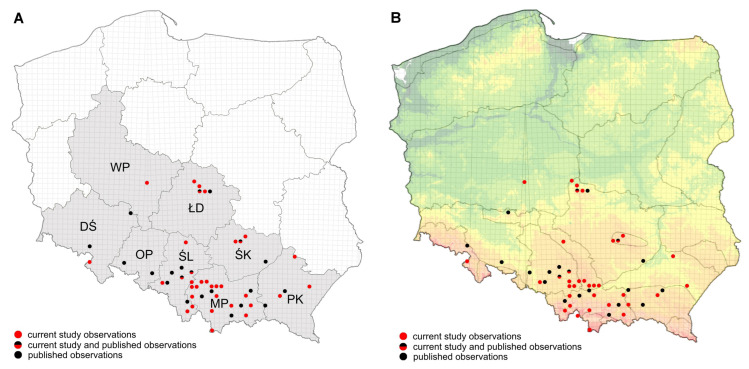
Observations of *Aedes japonicus* in Poland (2022–2025), according to voivodeship (**A**); and on physical map of Poland (**B**). Legend: DŚ—Dolnośląskie voivodeship, ŁD—Łódzkie voivodeship, MP—Małopolskie voivodeship, OP—Opolskie voivodeship, PK—Podkarpackie voivodeship, ŚK—Świętokrzyskie voivodeship, ŚL—Śląskie voivodeship, WP—Wielkopolskie voivodeship.

To generate the updated distribution map of *Aedes japonicus*, both the current dataset (67 observations) and previously published data [18,19] comprising 24 observations were used, yielding a total of 91 documented records from Poland between October 2022 and September 2025. The number of plotted points on the map (Figure 3) is lower, since some UTM grid squares contained multiple sites where the species was recorded. For example, in square CA56 (Śląskie Voivodeship), 13 observations were made (5 previously published, 8 in the present study) across four sites in Mikołów, two sites in the Katowice Ligota district, one site in the Katowice Kostuchna district, and one in the city center of Katowice. Similarly, square CC94 (Łódź) contained 8 observations from seven sites, DA24 (Kraków) contained 6 observations from six sites, and DB73 (Kielce) contained 5 observations from three sites. Kraków, Łódź, the Upper Silesian Industrial Region (Katowice and neighboring cities), as well as Kielce, represented urban/suburban areas where *Aedes japonicus* was most frequently observed and in the highest numbers of individuals (Supplementary Table S1).

## 4. Discussion

The results of the present study document 67 unpublished records of *Aedes japonicus* from Poland. This invasive species in Europe is currently found in half of the voivodeships, which represent the highest level of administrative division in Poland. Its wide distribution, as well as the high numbers observed at some sites, suggest that the introduction of the Asian bush mosquito into Poland must have taken place long before its first detection in 2022. Since this species shows both vector competence for West Nile virus in laboratory experiments [18] and has been identified as a WNV carrier in North American ecosystems [24,25], we conducted screening for the presence of the virus in field-collected *Ae. japonicus* in Poland. Our results are reassuring so far, as no genetic material of WNV was detected in 137 mosquitoes from three Polish sites where this species is found regularly and sometimes occurs in very high numbers. However, this situation may not be permanent, considering the pace of its expansive colonization pattern in Europe and the US. It is easy to imagine a scenario in which, as a dominant species, it could take over the role of native European mosquito species in transmitting WNV between animals and humans. It should be emphasized that the small sample size of our pilot screening study for the presence of WNV does not allow for drawing definitive conclusions about vector preference of *Aedes japonicus*. For these reasons, there is a continued need to monitor both the distribution of *Aedes japonicus* and the local populations of this mosquito species for WNV.

The presence of West Nile virus has been documented in dozens of mosquito species worldwide, including about 60 in North America [40]. In Europe, mosquitoes of the *Culex pipiens* species group are regarded as the main bridge vectors of WNV from birds to humans, as confirmed by a study conducted in Tierpark, Berlin, where WNV was found only in mosquitoes of this group [41]. It should be noted, however, that although the screening for WNV involved a representative sample of more than 2200 mosquitoes from at least seven species, it did not include *Aedes japonicus*. While the Asian bush mosquito is not currently considered a primary natural vector of WNV, under experimental conditions it exhibits higher transmission rates than mosquitoes from the *Culex pipiens* group [42]. In natural settings, however, pathogen transmission by vectors depends not only on experimentally determined competence, but above all on the intensity of interactions between the vector (mosquito), the virus-infected host (bird), and the pathogen (virus), within the specific characteristics of the local environment. With regard to WNV transmission by *Ae. japonicus*, its feeding preferences seem crucial. Studies on the blood meal composition of *Ae. japonicus* in the United States indicate that adult females primarily feed on the blood of wild mammals and humans [43,44]. In laboratory conditions, this species can also feed on bird blood [45,46]. The lack of bird blood in blood meals collected from mosquitoes in the US may, however, result from non-representative field samples or from local ecological factors, including the greater availability of mammals as hosts in the areas where the studies were conducted. Nevertheless, since WNV has been detected in field-collected *Ae. japonicus* in the US, there is no doubt that bird blood also serves as a food source for this species. Feeding preferences of *Ae. japonicus* in Europe may differ somewhat [47]. Interesting insights come from blood meal studies of mosquitoes collected at Zurich Zoo (Switzerland) [48], which for the first time documented the presence of bird blood in free-living *Ae. japonicus*. About 17% of captured specimens fed exclusively on bird blood, despite the abundant availability of mammalian hosts. This was also the only species in which the study demonstrated simultaneous feeding on both birds and mammals [48]. These findings may support the hypothesis that feeding preferences largely depend on host availability in the environment, and they demonstrate the high ecological plasticity of not only larvae but also adult *Ae. japonicus*.

The results of the present study indicate that in Poland, the Asian bush mosquito is most frequently observed in suburban areas (suburban habitats, 45.2% of observations), single-family housing estates, allotment gardens, cemeteries, as well as suburban and urban green areas such as forests, parks, or nature reserves adjacent to cities (forest/shrub habitats, 24.2% of observations). Such environments, located at the “interface of nature and civilization”, are rich both in human-made artificial containers used by mosquitoes during larval development and in sufficient populations of animals—wild as well as humans, their pets, and livestock (horse farms, hobby farms)—to ensure the survival of adult mosquitoes and the persistence of the species. These results are consistent with literature data, according to which *Ae. japonicus* prefers forested and shrubby habitats in rural, suburban, and urban areas [20,49]. According to current knowledge, *Aedes japonicus* does not show a preference for highly urbanized areas, unlike *Aedes albopictus* (Skuse, 1895) [50,51,52]. Also in a previous study by Schaffner et al. [19], which included 21 sampling sites in the southern part of Poland, as many as 71.4% of the sites were located in suburban areas. The remaining 28.6% of *Aedes japonicus* sites were distributed across other types of land use (urban habitat, agricultural areas, forest and shrub, and rural habitat). The observed differences may result, first, from variations in habitat classification and from ecological differences in the observation sites used in the two studies. Our findings also indicate that the peak occurrence of adult mosquitoes in Poland falls at the turn of summer and autumn, which confirms earlier observations from North America and Europe [20,46,52,53,54,55,56]. Given the relatively limited number of observations to date and the incomplete knowledge of the issue, further research on the ecology of *Aedes japonicus* in Poland is warranted.

The topic of a separate but closely related consideration—strongly linked to the climatic conditions prevailing in a given season—remains the temporal overlap between the peak occurrence of vectors and the amplification of West Nile virus within local ecosystems. Both mosquito abundance and WNV replication depend largely on temperature, while WNV amplification is highly heterogeneous over time and across seasons, in both Europe and the United States [24,33,57,58,59,60,61,62]. Some years are characterized by an increased number of outbreaks in animals and humans, interspersed with seasons of low viral transmission. There is evidence that warmer winter periods preceding seasons with high infection rates are an important factor influencing mosquito abundance as well as their vector competence [62,63,64]. This factor may be of particular significance in temperate climate countries such as Poland, Germany, the Czech Republic, and Slovakia, where no endemic outbreaks of the disease were recorded as recently as the 20th century. However, the climatic conditions in Central Europe have undergone substantial changes over the past several decades, contributing to the northward expansion of WNV.

In 2024—the year during which material for the present study was collected—the mean annual air temperature in Poland was 10.9 °C, exceeding the 1991–2020 reference period average by 2.2 °C [65,66]. The year 2024 was not only the warmest on record in Poland but also featured a relatively mild January, with a mean air temperature of −0.3 °C (0.9 °C higher than the reference period), an exceptionally warm February (5.7 °C, 5.8 °C above the 1991–2020 mean), and a warm March (6.7 °C, 3.6 °C above average) [67,68,69]. It is therefore not surprising that Poland experienced in 2024 the largest recorded outbreak of West Nile fever among wild birds. In Warsaw, a massive die-off of hooded crows (*Corvus corone cornix*, Linnaeus 1758) was once again observed—so substantial that the local population of this species within the monitored area was decimated [70]. These observations are consistent with data from the 2024 ECDC report [33]. According to this, human WNF cases in Europe were recorded between June and December, with a peak in incidence (>100 cases per week) between epidemiological weeks 30 (August) and 36 (September), roughly corresponding to data for the 2014–2018 period [57]. Since mosquito population dynamics—and consequently pathogen transmission—depend closely on climatic factors, the duration of the WNV season and the infection peak in 2024 varied geographically across European countries. The earliest, June cases were recorded in southern European countries with warmer climates, such as Greece and Spain, while the longest WNV transmission seasons occurred in Greece (June–December) and Germany (July–December). In Greece, December cases are well documented and unsurprising, given the confirmed presence of WNV in overwintering *Culex pipiens* populations [71], suggesting possible year-round viral circulation in that country. In contrast, in Germany, it is plausible that these late, isolated human cases may not represent endemic transmission.

Returning to Poland, in 2024, no genetically confirmed human WNF cases were reported. Outbreaks among horses and wild birds were observed between July and October, with the peak incidence occurring in October [33]. In neighboring Germany, where more than 250 WNV outbreaks in animals were confirmed, the veterinary case season lasted from July to November, with the highest incidence recorded in September [33].

In North America, *Ae. japonicus* has been recognized as a bridge vector of WNV. Considering European findings, along with the facts that this species is active throughout the WNV transmission season, shows strong territorial expansion [6,22,72], and is capable of dominating colonized habitats [22,23,72,73], it should also be regarded as an important candidate for a bridge vector, alongside *Culex pipiens*, transmitting WNV from avian hosts to humans in Europe [48].

## 5. Conclusions

Pilot screening for West Nile virus carried out on a sample of 137 free-living *Aedes japonicus* individuals collected from three locations in Poland did not reveal the presence of WNV genetic material in the mosquitoes. In 2025, the distribution of *Aedes japonicus* covers half of the voivodeships, which represent the highest level of administrative division in Poland. Most records come from the Małopolskie, Śląskie, and Łódzkie voivodeships. The most numerous adult populations were observed in the green areas of the city of Kielce (Świętokrzyskie voivodeship). The ecological preferences of *Ae. japonicus* in Poland are consistent with data from other European and US populations: larvae prefer human-made (artificial) containers for development; adults are most often observed in suburban areas; and peak occurrence/maximum number of records falls at the turn of summer and autumn. It should be noted, however, that the relatively limited number of *Aedes japonicus* observations to date justifies further research on the ecological preferences of this species in Poland. Undoubtedly, the invasive biology of *Aedes japonicus* necessitates also continuous monitoring of its distribution and the abundance of local populations, and warmer winters should be a warning signal, intensifying this research. It should be emphasized that the small sample size used in the present pilot screening study for the presence of WNV does not allow for drawing definitive conclusions regarding the vector competence of *Aedes japonicus*. Therefore, there is a continued need to study local populations of this mosquito species for WNV. Future studies on the vector competence of *Ae. japonicus* should be conducted on a significantly larger sample of mosquitoes, covering August and September, preferably in areas of local West Nile fever outbreaks among birds and horses.

The results of the present study, similarly to other work on invasive mosquito species [74,75], confirm the usefulness of data collected by naturalists via the iNaturalist platform as a supplementary source of information supporting vector monitoring, though one that requires verification.

## Figures and Tables

**Table 1:** The number of *Aedes japonicus* specimens caught in individual locations.

Date	Number of *Ae. japonicus* Specimens Caught (Collectors)			
	Kielce	Mikołów	Barcza	Kraków
1 September 2024	72 (P.N., K.B.)			
4 September 2024		9 (P.N., M.G.)		
11 September 2024		18 (P.N., M.G.)		
12 September 2024			20 (K.B.)	
18 September 2024		6 (M.G.)		
24 September 2024				12 (Ł.M.)

**Table 2:** Number of *Aedes japonicus* observations in individual voivodeships, depending on the stage of development and land use.

Voivodeship	Number of Observations			Observations (n) of Larvae/Adults in the Context of Land Use			
	All	Larvae	Adults	Forest/Shrub	Rural	Suburban	Urban
Dolnośląskie	1	0	1	0	0	0/1	0
Łódzkie	14	3	11	0/1	0	2/8	1/2
Małopolskie	21	3	20	1/7	2/3	0/3	0/7
Podkarpackie	7	2	7	0	2/5	0/2	0
Śląskie	17	1	16	1/2	0	0/13	0/1
Świętokrzyskie	6	0	6	0/5	0	0/1	0
Wielkopolskie	1	0	1	0	0/1	0	0
Total	67	9	62	2/15	4/9	2/28	1/10

## Data Availability

All data were made available in the paper and in the Table S1.

## Supplementary Materials

The following supporting information can be downloaded at: https://www.mdpi.com/article/10.3390/v17111515/s1, Table S1: *Aedes japonicus* distribution in Poland.

## Abbreviations

The following abbreviations are used in this manuscript:

*Ae.*	*Aedes*
ECDC	European Centre for Disease Prevention and Control
ELISA	Enzyme-linked immunosorbent assay
RNA	Ribonucleic acid
RT-PCR	Reverse Transcription Polymerase Chain Reaction
rRT-PCR	real-time Reverse Transcription Polymerase Chain Reaction
UTM	Universal Transverse Mercator
WNF	West Nile fever
WNV	West Nile virus
